# *In Vitro* and *In Vivo* Evidence that Thrombospondin-1 (TSP-1) Contributes to Stirring- and Shear-Dependent Activation of Platelet-Derived TGF-β1

**DOI:** 10.1371/journal.pone.0006608

**Published:** 2009-08-12

**Authors:** Jasimuddin Ahamed, Christin A. Janczak, Knut M. Wittkowski, Barry S. Coller

**Affiliations:** Laboratory of Blood and Vascular Biology, The Rockefeller University, New York, New York, United States of America; University of Giessen Lung Center, Germany

## Abstract

Thrombospondin 1 (TSP-1), which is contained in platelet α-granules and released with activation, has been shown to activate latent TGF-β1 *in vitro*, but its *in vivo* role is unclear as TSP-1-null (*Thbs1^−/−^*) mice have a much less severe phenotype than TGF-β1-null (*Tgfb1^−/−^*) mice. We recently demonstrated that stirring and/or shear could activate latent TGF-β1 released from platelets and have now studied these methods of TGF-β1 activation in samples from *Thbs1^−/−^* mice, which have higher platelet counts and higher levels of total TGF-β1 in their serum than wild type mice. After either two hours of stirring or shear, *Thbs1^−/−^* samples demonstrated less TGF-β1 activation (31% and 54% lower levels of active TGF-β1 in serum and platelet releasates, respectively). TGF-β1 activation in *Thbs1^−/−^* mice samples was normalized by adding recombinant human TSP-1 (rhTSP-1). Exposure of platelet releasates to shear for one hour led to near depletion of TSP-1, but this could be prevented by preincubating samples with thiol-reactive agents. Moreover, replenishing rhTSP-1 to human platelet releasates after one hour of stirring enhanced TGF-β1 activation. *In vivo* TGF-β1 activation in carotid artery thrombi was also partially impaired in *Thbs1^−/−^* mice. These data indicate that TSP-1 contributes to shear-dependent TGF-β1 activation, thus providing a potential explanation for the inconsistent *in vitro* data previously reported as well as for the differences in phenotypes of *Thbs1^−/−^* and *Tgfb1^−/−^* mice.

## Introduction

Transforming growth factor β1 (TGF-β1) is a multifunctional cytokine that plays an important role in regulating immune response, cell proliferation, angiogenesis, wound healing, and tissue fibrosis[1]–[3]. Blood platelets contain 40−100 times as much TGF-β1 as other cells[4] and release it when activated by a variety of agents, including thrombin[5]–[11]. However, virtually all TGF-β1 released from platelets is in an inactive multicomponent complex [large latent complex (LLC)] in which TGF-β1 is noncovalently bound to latency-associated peptide (LAP), which, in turn, is disulfide bonded to latent TGF-β binding protein-1 (LTBP-1)[12], [13].

*In vitro* studies have used multiple methods to activate latent TGF-β1, including exposure to proteases, thrombospondin-1 (TSP-1), reactive oxygen species, and binding to integrin receptors[7], [8], [10], [13]–[26], but the mechanism of *in vivo* activation remains unclear. Recently, we have shown that latent TGF-β1 released from human platelets or skin fibroblasts can be activated through stirring or shear force[12] and that thiol-disulfide exchange contributes to this process.

Support for a role for TSP-1 in TGF-β1 activation *in vivo* comes from studies of TSP-1-null (*Thbs1^−/−^*) mice, which demonstrate pathological changes similar to those of TGF-β1-null (*Tgfb1^−/−^*) mice[27], [28] although the latter have a much more severe phenotype [29], [30] usually dying within weeks after birth. To gain additional insights into the effects of TSP-1 on TGF-β1 activation, we studied the effects of stirring and shear on latent TGF-β1 activation in WT and *Thbs1^−/−^* mice both *in vitro* and *in vivo*. We focused on the activation of TGF-β1 released from platelets because TGF-β1 and TSP-1 are both highly concentrated in platelet α-granules and released with platelet activation[19], [31]–[35].

## Results

### TSP-1-null mice have more platelets than WT mice

Mean platelet volumes (MPV) and hematologic parameters were similar in WT and *Thbs1^−/−^* mice except that *Thbs1^−/−^* mice (n = 16) had approximately 22% higher platelet counts than WT mice (n = 16; p<0.005) (**Table 1**).

**Table 1:** WT, wild type; *Thbs1^−/−^*, TSP-1-null; WBC, white blood count; RBC, red blood cell count; HCT, hematocrit; MCV, mean corpuscular volume; PLT, platelets; MPV, mean platelet volume; fL, femtoliters.

		WBC (x10ˆ6 cells/mL)	Neutrophil (% of WBC)	Lymphocyte (% of WBC)	Monocyte (% of WBC)	Eosinophil (% of WBC)	RBC (x10ˆ9 cells/mL)	HCT (%)	MCV (fL)	PLT (x10ˆ6 cells/mL)	MPV (fL)
WT (n = 16)	MEAN (SD)	6.6 (2.3)	7.5 (4.0)	84.0 (4.0)	1.6 (1.4)	4.6 (2.0)	9.7 (1.1)	47.4 (7.0)	48.0 (3.7)	1214.0 (170)	5.8 (0.8)
Thbs1-/- (n = 16)	MEAN (SD)	8.2 (3.7)	8.3 (4.0)	84.0 (5.4)	2.1 (1.3)	3.7 (1.7)	9.9 (0.8)	48.0 (6.4)	47.4 (4.4)	1480.0 (300)*	5.6 (0.8)

* p<0.005 vs WT.

### TSP-1 contributes to stirring- or shear-mediated TGF-β1 activation in serum

**Figure 1:**
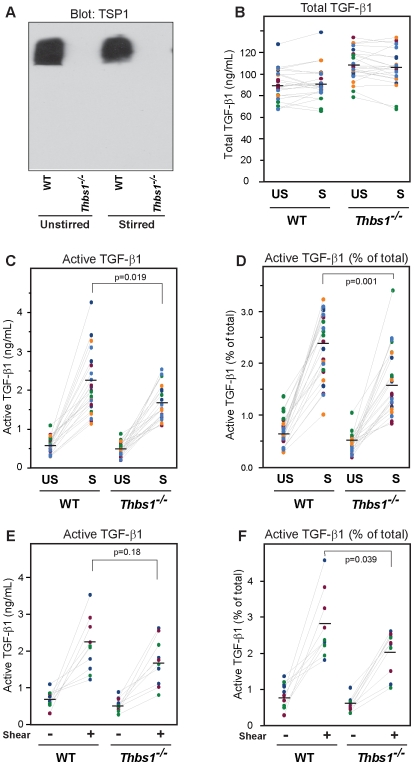
Sera from *Thbs1^−/−^* mice have reduced ability to undergo activation of TGF-β1 by stirring or shear. (A) Immunoblots of WT and *Thbs1^−/−^* mice sera demonstrate absence of TSP-1 in the *Thbs1^−/−^* mice. (B, C, D) Sera from WT (n = 23) and *Thbs1^−/−^* (n = 23) mice were stirred (S) at 1,200 rpm or left unstirred (US) for 120 min at 37°C and then total (B) and active (C, D) TGF-β1 were measured; the latter was expressed either as an absolute value (ng/mL) (C) or as a percentage of total TGF-β1 (D). Levels of active TGF-β1 increased less in *Thbs1^−/−^* than WT mice with stirring [p = 0.057 (absolute values) and p = 0.016 (percentages of total TGF-β1) for interaction by ANOVA]. The post-stirring values were also higher in WT than *Thbs1^−/−^* mice [p = 0.19 (absolute values) and p = 0.001 (percentages of total TGF-β1) by t-test]. (E, F) Sera from WT (n = 10) or *Thbs1^−/−^* (n = 10) mice were either incubated at 37°C (−) or subjected to shear (+) at 1,800 s^−1^ at 37°C for 120 min. Active TGF-β1 increased more in WT mice, both in terms of absolute values (p = 0.18 by t-test) (E) and as percentages of total TGF-β1 (p = 0.039 by t-test) (F).

To assess whether TSP-1 contributes to stirring-dependent activation of latent TGF-β1, serum samples were obtained from WT and *Thbs1^−/−^* mice on five days. Immunoblotting confirmed that the sera of *Thbs1^−/−^* mice lack TSP-1 protein (**Fig. 1A**). Each sample was divided and incubated at 37°C for 2 hours with or without stirring at 1,200 rpm.

In unstirred serum, total TGF-β1 levels were approximately 19% higher in *Thbs1^−/−^* mice than in WT mice (**Fig. 1B**) [91±15 ng/mL in WT (n = 23) and 108±15 ng/mL in *Thbs1^−/−^* mice (n = 23); p<0.001]. Higher serum levels of TGF-β1 in *Thbs1^−/−^* mice are consistent with their higher platelet counts since plasma levels of TGF-β1 are only approximately 2–4 ng/mL and nearly all of serum TGF-β1 is released from platelets during clot formation.

Stirring of WT or *Thbs1^−/−^* sera for 2 hours had little impact on total TGF-β1 levels (**Fig. 1B**), but increased levels of active TGF-β1 more in WT sera than *Thbs1^−/−^* sera when expressed either as absolute values or as percentages of total TGF-β1 (**Fig. 1C, D**) [absolute values increased from 0.5 to 2.2 ng/mL in WT mice (n = 23) and from 0.6 to 1.6 ng/mL in *Thbs1^−/−^* mice (n = 23; p = 0.057 for interaction by ANOVA); values expressed as percentages of total TGF-β1 increased from 0.7 to 2.3% in WT mice (n = 23) and from 0.5 to 1.6% in *Thbs1^−/−^* mice (n = 23; p = 0.016 for interaction by ANOVA)]. The final values of active TGF-β1 were higher in WT mouse samples than in *Thbs1^−/−^* samples (**Fig. 1C, D**)

Similar results were obtained when sera from WT and *Thbs1^−/−^* mice were subjected to shear for 2 hours in a cone and plate device. The differences in final values in this smaller sample were not statistically significant when expressed as absolute values [active TGF-β1 was 2.2±0.7 ng/mL in WT mice (n = 10) and 1.7±0.6 ng/mL in *Thbs1^−/−^* mice (n = 10) (p = 0.18 by t-test)], but were significant when expressed as percentages of total TGF-β1 [active TGF-β1 2.7±0.8% in WT mice (n = 10) and 2.0±0.6% in *Thbs1^−/−^* mice (n = 10) (p = 0.039 by t-test)]. In the combined sample, the differences in increases between control and either stirred or sheared sera were greater in WT (n = 33) than *Thbs1^−/−^* mice (n = 33) with respect to both absolute values (p = 0.4) and percentages of total TGF-β1 (p = 0.01) **(** **Fig. 1E, F** **)**.

### TSP-1 contributes to stirring-dependent activation of TGF-β1 in platelet releasates

**Figure 2:**
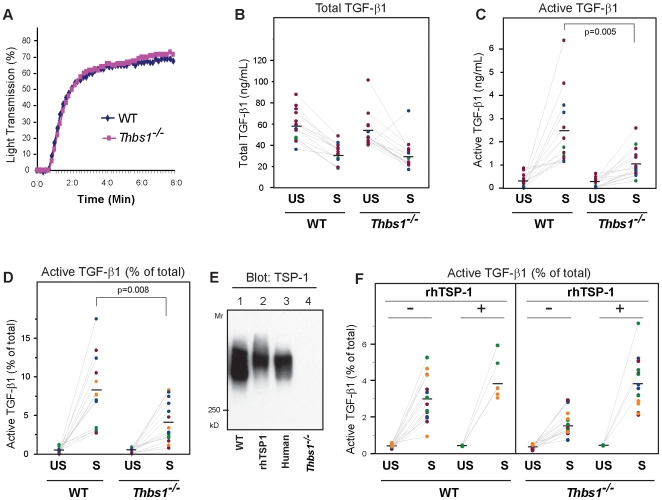
Stirring increases TGF-β1 activation in platelet releasates from WT mice more than from *Thbs1* *^−^* ^*/−*^ mice and recombinant human TSP-1 corrects the defect. (A) Thrombin-induced aggregation of washed platelets from WT and *Thbs1^−/−^* mice. (B, C, D) Platelet releasates were either left unstirred (US) at 37°C or stirred (S) at 1,200 rpm for 120 min and then total (B) and active (C, D) TGF-β1 were measured. (B) The decline of total TGF-β1 in stirred versus unstirred platelet releasates was similar in WT and *Thbs1^−/−^* mice. (C, D) Active TGF-β1 increased more in WT than *Thbs1^−/−^* mice [p = 0.004 (absolute values) and p = 0.005 (percentages of total TGF-β1) for interaction by ANOVA]. The final values were also higher in WT vs *Thbs1^−/−^* mice (p = 0.005 and p = 0.008 by t-test). (E) Immunoblotting of platelet releasates obtained from WT or TSP-1 null mice demonstrated absence of TSP-1 protein in a *Thbs1^−/−^* mouse (lane 4); addition of 20 µg/mL of rhTSP-1 protein to the *Thbs1^−/−^* mouse sample (lane 2) achieved a TSP-1 level similar to those in a WT mouse sample (lane 1) and a human platelet releasate (lane 3). (F) Adding rhTSP-1 (+) versus BSA (−) corrected the defect in stirring-induced activation of TGF-β1 in *Thbs1^−/−^* mice.

Similar experiments were conducted with thrombin-stimulated platelet releasates. Thrombin-induced platelet aggregation was similar in WT and *Thbs1^−/−^* mice (**Fig. 2A**). Unlike in serum samples, total TGF-β1 values in platelet releasates after thrombin stimulation were similar in WT and *Thbs1^−/−^* mice [58±14 ng/mL in WT mice (n = 14) and 53±16 ng/mL in *Thbs1^−/−^* mice (n = 14)], consistent with the adjustment of the platelet counts in the washed platelet preparations to the same level in both WT and *Thbs1^−/−^* mice. As we previously observed with human platelet releasates, total TGF-β1 from WT and *Thbs1^−/−^* mice platelet releasates decreased over 2 hours of stirring by approximately 40−50%, possibly due to adsorption to the wall of the cuvette and/or the stir bar[12]; similar decreases in total TGF-β1 were observed with platelet releasates from *Thbs1^−/−^* mice (**Fig. 2B**). Basal levels of active TGF-β1 in the unstirred samples were also similar in WT and *Thbs1^−/−^* mice [0.3±0.3 ng/mL in WT mice (n = 14) and 0.2±0.2 ng/mL in *Thbs1^−/−^* mice (n = 14)] while post-stirring values were lower in *Thbs1^−/−^* mice [absolute values: 1.1±0.6 ng/mL versus 2.4±1.5 ng/mL versus (p = 0.005); percentages of total TGF-β1: 4.0±2.6% versus 8.0±4.2% (p = 0.008)]. Taken together, stirring increased active TGF-β1 more in WT (n = 14) than *Thbs1^−/−^* mice (absolute values: p = 0.004, percentage of total TGF-β1: p = 0.005 for interaction by ANOVA) (**Fig. 2C, D**). A difference between WT and *Thbs1^−/−^* mice was also observed when platelet releasates were stirred for 30 min [active TGF-β1 increased from 0.10±0.015% to 4.40±1.30% of total TGF-β1 in WT versus 0.10±0.010% to 2.80±0.55% of total TGF-β1 in *Thbs1^−/−^* mice (n = 3)].

To further assess whether the defect in TGF-β1 activation by stirring in *Thbs1^−/−^* mice is due to the loss of TSP-1 protein, we tested whether purified recombinant human TSP-1 (rhTSP-1) could correct the defect in the *Thbs1^−/−^* mice samples. Preliminary immunoblot studies indicated that adding 20 µg/ml of rhTSP-1 to platelet releasates of *Thbs1^−/−^* mice produced TSP-1 levels comparable to those in platelet releasates from WT mice and humans (**Fig. 2E**). Adding rhTSP-1 to unstirred platelet releasates from both WT and *Thbs1^−/−^* mice did not change total or active TGF-β1 [total TGF-β1 in control unstirred samples was 48±8 ng/mL in WT mice (n = 15) and 45±6 ng/mL in *Thbs1^−/−^* mice (n = 15) and after adding rhTSP-1 the values were 48±6 ng/mL in WT mice (n = 4) and 44±3 ng/mL in *Thbs1^−/−^* mice (n = 4)]. Adding rhTSP-1 to WT platelet releasates had only a minor effect on stirring-induced activation of TGF-β1 (**Fig. 2F**). In sharp contrast, adding rhTSP-1 to platelet releasates from *Thbs1^−/−^* mice fully corrected the defect in stirring-induced activatibility (**Fig. 2F**).

Since previous studies indicated that the peptides VLAL and LSKL can block TSP-1-mediated activation of the TGF-β1 latent complex[36], [37], we studied their effects on stirring-dependent TGF-β1 activation. Adding either VLAL or LSKL (100 µM) failed to inhibit stirring-induced TGF-β1 activation in human platelet releasates when compared to a control peptide, SLLK (stirring increased active TGF-β1 from 0.25±0.05% to 5.1±1.9% of total TGF-β1 in samples with SLLK, from 0.25±0.04% to 5.2±1.6% in samples with VLAL, and from 0.16±0.12% to 5.3±1.8% in samples with LSKL). An activating peptide KRFK also had little or no effect on stirring-dependent TGF-β1 activation (data not shown)

### Shear depletes TSP-1 from human platelet releasates via a thiol-dependent mechanism

**Figure 3:**
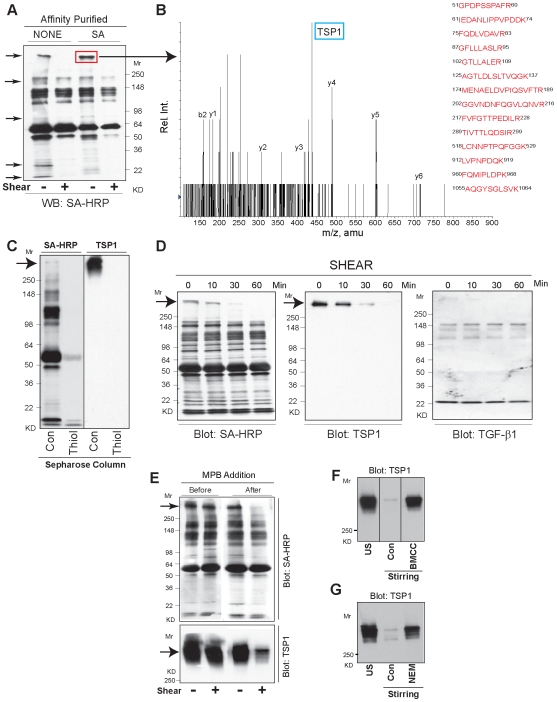
Shear depletes TSP-1 via a thiol-dependent mechanism. (A) The proteins in human platelet releasates were labeled with MPB (100 µM) for 30 min either before (−) or after (+) shear for 2 hours. The labeled proteins were either analyzed directly (left two lanes) or after affinity-purification using Streptavidin-coupled beads (right two lanes). Shearing led to a dramatic decrease in intensity of the HRP reaction in select regions. (B) One of the MPB-labeled proteins (boxed) that was most affected by shearing was identified as TSP-1 by LC-MS/MS analysis. (C) Platelet releasates were passed through either a control-Sepharose column (Con) or a thiol-Sepharose column (Thiol) and then labeled with MPB. Depletion of thiol-reactive proteins by the column was analyzed by reaction of the separated proteins with Streptavidin (left panel) and depletion of TSP-1 protein was measured by immunoblotting with an anti-TSP-1 antibody (right panel). Nearly all of the proteins that labeled with MPB from the control column were not labeled after passage through the thiol-Sepharose column. (D) Effect of increasing time of exposure to shear on depletion of TSP-1 from platelet releasates. MPB labeling of TSP-1 was concordantly reduced with the loss to TSP-1 protein during shear as judged by reaction with Streptavidin-HRP (left panel) and immunoblotting with an anti-TSP-1 antibody (middle panel). TGF-β1 depletion was much less pronounced as judged by immunoblotting with an anti-TGF-β1 antibody (right panel). (E) Addition of MPB (100 µM) before shear partially prevented the loss of TSP-1 protein as shown by immunoblotting with an anti-TSP-1 antibody. Addition of the other thiol-reactive reagents, BMCC (F) or NEM (G), similarly protected against loss of TSP-1. Vertical lines in (F) indicate deletion of intermediate lanes from the same gel.

Since thiol-disulfide exchange contributes, at least in part, to shear-induced TGF-β1 activation[12] we tested whether the TSP-1-mediated contribution involves thiol-disulfide exchange. As we previously reported, when MPB was added to human platelet releasates before shear, it labeled a number of proteins as judged by reaction with enzyme-linked Streptavidin (SA) SA-HRP (**Fig. 3A**). The enzymatic activity intensity was markedly diminished, however, in several regions including the one corresponding to TGF-β1 band when MPB was added after shear[12]. The enzymatic activity corresponding to a band that migrated at Mr >300 kD under nonreducing conditions was dramatically reduced after shear (**Fig. 3A** boxed area). To identify the protein in the unstirred sample, it was purified with SA-coupled beads and analyzed by SDS-PAGE. The band was excised, subjected to trypsin digestion and analyzed by mass spectrometry. Fourteen peptides identified by mass spectrometry corresponded to sequences found in TSP-1 (**Fig. 3B**).

To assess the percentage of TSP-1 molecules that contain accessible sulfhydryl groups, unstirred platelet releasates were subjected to affinity chromatography on either a control-Sepharose column or a thiol-Sepharose column. The eluates were analyzed for the presence of proteins containing free thiols by MPB labeling and TSP-1 by immunoblotting (**Fig. 3C**). Nearly all of the thiol-reactive proteins bound to the thiol-Sepharose column (as judged by the decrease in MPB labeling), whereas there was little or no binding of thiol-reactive proteins to the control Sepharose column.

We tested whether TSP-1, like TGF-β1, becomes depleted from platelet releasates when subjected to shear. We found a time-dependent loss of TSP-1 protein as judged by immunoblot analysis (**Fig. 3D**, center panel), which could fully account for the reduction in thiol-labeling at the expected Mr of the TSP-1 band (**Fig. 3D**, left panel). TSP-1 depletion was nearly complete within 1 hour (**Fig. 3D** center panel), and was greater than the partial depletions of the Mr 25 kD TGF-β1 band (**Fig. 3D**, right panel) and the major LAP (Mr 270 kD) band (data not shown). Adding MPB before shear partially prevented the loss of TSP-1 protein (**Fig. 3E**), as did the other thiol-reactive reagents BMCC (**Fig. 3F**) and N-ethylmaleimide (NEM) (**Fig. 3G**). Densitometric quantitation of TSP-1 bands from three independent experiments showed that adding NEM before shear reduced TSP-1 protein depletion during shear by approximately 50% (data not shown). Thus, shear-dependent loss of TSP-1 appears to require free thiols.

### Replenishing TSP-1 after stirring-induced depletion enhances TGF-β1 activation

Since we observed both depletion of TSP-1 and a plateau of TGF-β1 activation after 1 hour of stirring, we hypothesized that this plateau may be due to TSP-1 depletion. To test this hypothesis, we performed experiments in which we added either exogenous rhTSP-1 or buffer after 1 hour of stirring and then continued the stirring for an additional 1 hour. Adding rhTSP-1 further increased the activation of TGF-β1 by approximately 37% compared to samples treated with buffer [from 4.8% to 7.6% of total TGF-β1, SD = 0.9% (p<0.001 by paired t-test, n = 6)].

### More active TGF-β1 can be recovered over time from platelet-rich thrombi formed *in vivo* from WT than TSP-1-null mice

**Figure 4:**
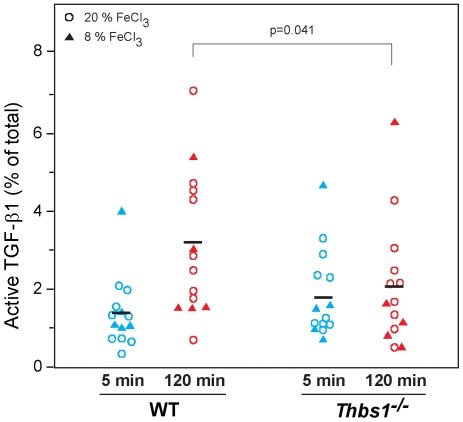
TSP-1 is required for *in vivo* time-dependent activation of TGF-β1 in platelet-rich thrombi. Thrombosis was induced in the carotid arteries of WT or *Thbs1^−/−^* mice by exposure to either 8% FeCl_3_ (filled triangles, n = 20) or 20% FeCl_3_ (open circles, n = 36) for 3 min. At either 5 min or 120 min after the vessel became completely occluded, approximately 4 mm carotid arterial segments were excised. The thrombi from the vessels were removed and extracts from them were prepared for analysis of active and total TGF-β1. Since the effects of 20% and 8% FeCl_3_ were similar, the data were pooled. Active TGF-β1 levels expressed as a percentage of total TGF-β1 were similar after 5 min. The values of active TGF-β1 increased with time after occlusion in WT but not in *Thbs1^−/−^* mice (p = 0.0024 for interaction by ANOVA). Moreover, the values in the 120 min samples of WT mice were higher than those in *Thbs1^−/−^* mice (p = 0.041 by t-test).

After exposure to 20% ferric chloride (FeCl_3_), thrombi formed rapidly in the carotid arteries of WT and *Thbs1^−/−^* mice, resulting in complete and stable occlusion of the vessels after 5.2±0.9 min in 18 of 20 WT mice and 5.5±0.8 min in all 18 *Thbs1^−/−^* mice. The amounts of total TGF-β1 extracted in the buffer from the thrombi contained in the arterial segments of WT and *Thbs1^−/−^* mice were similar when the vessels were removed 5 min after total occlusion of the artery [5.6±2.4 ng/mL in WT mice (n = 9) and 5.5±1.6 ng/mL in *Thbs1^−/−^* mice (n = 9)]. The levels of active TGF-β1 expressed as percentages of total TGF-β1 were higher after 120 min than after 5 min of total occlusion in WT mice, but not in *Thbs1^−/−^* mice [1.1±0.6% after 5 min (n = 9) and 3.4±1.9% after 120 min (n = 9) in WT mice; 1.8±0.9% after 5 min (n = 9) and 2.0±1.1% after 120 min (n = 9) in *Thbs1^−/−^* mice, p = 0.0024 for interaction by ANOVA] (**Fig. 4** **, open circles**). The concentrations of active TGF-β1 in the extracts obtained after 120 min of occlusion were also higher in WT mice than *Thbs1^−/−^* mice (p = 0.041 by t-test).

When 8% FeCl_3_ was used instead of 20%, similar results were obtained, but a higher percentages of thrombi were not stable throughout the experiment. Thus, complete and stable occlusion of the vessel occurred after 5.8±0.8 min in only 10 of 15 WT mice and 7.1±0.8 min in only 10 of 12 *Thbs1^−/−^* mice. Mice that did not form stable thrombi were excluded from the analysis. The amounts of total TGF-β1 in the thrombi removed 5 min after total occlusion in WT and *Thbs1^−/−^* mice were similar [4.0±2.6 ng/mL in WT mice (n = 5) and 3.9±2.1 ng/mL in *Thbs1^−/−^* mice (n = 5)]. Levels of active TGF-β1, expressed as a percentage of total TGF-β1 were higher in extracts prepared from thrombi obtained after 120 min of occlusion than after 5 min in WT mice, but not in *Thbs1^−/−^* mice [1.7±1.2% after 5 min (n = 5) and 2.6±1.7% after 120 min (n = 5) in WT mice; 1.9±1.6% after 5 min (n = 5) and 2.0±2.3% after 120 min (n = 5) in *Thbs1^−/−^* mice] (**Fig. 4** **, filled triangles**).

Since the effects of 20% and 8% FeCl_3_ were similar (**circles vs triangles,** **Fig. 4**), we analyzed the pooled data. Active TGF-β1, expressed as a percentage of total TGF-β1, was higher after 120 min of occlusion than after 5 min in WT mice, but not in *Thbs1^−/−^* mice (**Fig. 4**) [1.3±0.9% after 5 min and 3.1±1.8% after 120 min in WT mice; 1.8±1.2% after 5 min and 2.0±1.6% after 120 min in *Thbs1^−/−^* mice (p = 0.0024 for interaction by ANOVA)].

By comparing the αIIb immunoblot intensities from known numbers of washed murine platelets to those obtained with the lysates of thrombi, we estimated that approximately 0.6×10^8^ platelets are present in each carotid artery thrombus. We previously found approximately 10 ng of total TGF-β1 per 10^8^ platelets[12], which is consistent with the approximately 8 ng of total TGF-β1 we recovered per thrombus being due to TGF-β1 release from platelets in the thrombi.

## Discussion

Our data indicate that TSP-1 contributes to TGF-β1 activation in both serum and platelet releasates under conditions of stirring and/or shear. We found that TGF-β1 can become activated after being released from platelets *in vitro*, that the activation is reduced in the absence of TSP-1, and that full activation can be restored by reconstitution with purified TSP-1. Moreover, we found that TSP-1 becomes depleted from WT samples during stirring or shear, and that replenishing TSP-1 during stirring enhances the activation of TGF-β1. These results have implication for the role of TSP-1 in the physiologic and pathologic mechanism(s) of platelet-derived TGF-β1 activation and reconcile some of the paradoxical findings that have been reported previously.

We found that *Thbs1^−/−^* mice have higher numbers of platelets than WT mice and this can account for the higher serum levels of total TGF-β1 in *Thbs1^−/−^* mice than WT mice. Active TGF-β1 levels before stirring or shear are lower in sera from *Thbs1^−/−^* mice than WT mice, suggesting that even in the absence of stirring or shear, TSP-1 may make some contribution to TGF-β1 activation during the 4 hours of blood clotting used to prepare serum. The absolute differences are, however, very small. Stirring or exposure to shear forces accentuates the difference in TGF-β1 activation, with higher levels of active TGF-β1 being produced in WT than *Thbs1^−/−^* mice, whether expressed as an absolute value or as a percentage of total TGF-β1. Stirring or shear does, however, increase the level of active TGF-β1 in the serum of *Thbs1^−/−^*mice, demonstrating that activation is not entirely TSP-1 dependent. Similar data were obtained when platelet releasates from the two groups of mice were stirred, although the absolute values differed. Most importantly, the addition of rhTSP-1 to the *Thbs1^−/−^*mice samples fully corrected the defect in TGF-β1 activatability, providing strong support for the interpretation that the differences we observed with sera and platelet releasates are unlikely to be due to differences in proteins other than TSP-1.

Several observations support a biological role for TSP-1 in the activation of TGF-β1. Thus, *in vitro* studies have demonstrated that purified TSP-1 can activate TGF-β1 and the sites of interaction between the molecules have been localized to the K^412^RFK^415^ sequence between the first and second type 1 repeats of TSP-1 and the L^54^SKL^57^ sequence in LAP[38]. Moreover, there is evidence of an intimate association between the two molecules since TGF-β1 copurifies with TSP-1 and purified radiolabeled TGF-β1 can bind to TSP-1, presumably by interacting with the WSXW sequence in the type-1 repeats[19], [20], [22]–[24], [38]. Most importantly, although one must be cautious in making judgments about the importance of a protein based on the phenotype of gene targeted animals, the similarity in organ damage in *Tgfb1^−/−^*mice and *Thbs1^−/−^* mice provides support for an *in vivo* role for TSP-1 in TGF-β1 activation, especially since treating the *Thbs1^−/−^* mice with the KRFK peptide, partially rescued the pathological changes in the lungs and pancreas of *Tgfb1^−/−^*mice[27]. In addition, *Thbs1^−/−^* mice subjected to coronary occlusion and reperfusion show enhanced myocardial inflammation and adverse myocardial remodeling in association with a trend towards reduced TGF-β1 signaling relative to WT mice[39]. A role for TSP-1 in TGF-β1 activation may have particular significance for platelet biology since platelets contain high concentration of both TSP-1 and TGF-β1 and release both in response to thrombin[19], [20], [31]–[35].

On the other hand, the much greater severity of the phenotype of the *Tgfb1^−/−^*mice than the *Thbs1^−/−^* mice makes it clear that a mechanism(s) of TGF-β1 activation that does not require TSP-1 must exist *in vivo*. In addition, Grainger and Frow could not activate TGF-β1 contained in latent complexes with platelet-derived purified TSP-1[40], and Abdelouahed et al. found no evidence for TSP-1 activating TGF-β1 released from platelets, with both WT and *Thbs1^−/−^* mice having similar levels of active TGF-β1[5]. A number of technical differences in the design of the experiments we conducted and those conducted by these investigators can perhaps reconcile the apparent discrepancies in our results. Thus, Grainger and Frow appear to have incubated their reagents without stirring and Abdelouahed et al. added thrombin to washed platelets and stirred for only 3 min before assaying the supernatant. In contrast, we stirred samples (serum or platelet releasates) for 2 hours because our previous studies demonstrated that stirring- or shear-induced TGF-β1 activation is time-dependent and requires more than 1 hour to reach its plateau.

Since it has been reported that the TGF-β1 complex peptides VLAL and LSKL can inhibit TSP-1-mediated activation of TGF-β1 in some systems[36], [37], we tested the effects of these peptides on stirring-induced activation of TGF-β1 in platelet releasates. These peptides had little or no effect on TGF-β1 activation, suggesting that shear- and/or stirring-induced activation may involve interactions mediated by other regions of the TGF-β1 complex.

Our data indicate that under conditions of optimal stirring and shear, the presence of TSP-1 at levels found in serum and platelet releasates enhances TGF-β1 activation. The TSP-1 contribution in serum increases active TGF-β1 by approximately 0.5–1.0 ng/mL, which is consistent with a biologically important role, since this concentration is approximately 50- to 100-fold more than the minimum concentration required to induce transcription of the TGF-β1-responsive gene plasminogen activator inhibitor-1 (PAI-1)[41].

It is of interest to consider why the TSP-1 contribution to activation of TGF-β1 is limited to only approximately 1% of total serum TGF-β1. It has been suggested that post-translational modifications of one or both molecules may be required for complex formation[5]. Our data can be interpreted to suggest two additional hypotheses. The first is that only a subpopulation of TSP-1 molecules in the correct conformation(s) (regardless of post-translational modifications) can participate in the activation process. In fact, it has been demonstrated that TSP-1 adopts markedly different conformations, depending on the temperature, pH, cation concentrations, and degree of purification[42]–[45]. Support for this hypothesis comes from evidence that the conformation of TSP-1 affects its efficiency in mediating thiol-disulfide exchange with other molecules[42]–[44], [46]. We previously found that thiol-disulfide exchange contributes partially to the stirring- and shear-induced TGF-β1 activation mechanism and in this study we demonstrated that TSP-1 is a shear-sensitive protein and that free thiols contribute to the changes that TSP-1 undergoes when subjected to shear force. Moreover, shear has been found to facilitate thiol-disulfide exchange reactions in a number of systems, including modification of von Willebrand factor (vWf), one of the proteins demonstrated to undergo thiol-disulfide exchange in the presence of TSP-1[47], [48]. It is therefore possible that TSP-1-mediated TGF-β1 activation also requires thiol-disulfide exchange. The second hypothesis is that TSP-1 depletion limits the contribution of TSP-1 to TGF-β1 activation. This would provide a negative feedback control mechanism to prevent excessive TGF-β1 activation unless there was an ongoing supply of TSP-1 as may occur if there is continued release of TSP-1 from additional platelets. In fact, we found that replenishing TSP-1 during stirring results in a further increase in the TGF-β1 activation. These hypotheses are not mutually exclusive and thus multiple mechanisms may contribute to the observed phenomenon.

Our data may also bear on the interesting question of how high concentrations of both TGF-β1 and TSP-1 can be stored in platelet α-granule without inducing activation of TGF-β1. It has previously been hypothesized that an inhibitory molecule in α-granules may limit the activation of TGF-β1 by TSP-1[5]. Our finding that TSP-1 mediated activation requires stirring or shear provides an alternative explanation, as well as a mechanism by which TGF-β1 released from platelets along with TSP-1 can be activated by the shear forces in the circulation. In addition, if thiol-disulfide exchange is required for activation, the relatively low pH in α-granules[49] may limit activation because thiol-disulfide exchange is favored at higher than neutral pH where there is a higher ratio of protonated thiol than deprotonated thiolate group[12], [50].

In conclusion, TSP-1 contributes to stirring- and shear-induced TGF-β1 activation *in vitro* and to TGF-β1 activation over time in platelet-rich thrombi *in vivo*. These data provide an explanation for the presence of a mild *Tgfb1^−/−^*-like phenotype in *Thbs1^−/−^* mice. Our data also provide evidence that free thiols in TSP-1 affects its ability to undergo stirring- and shear-dependent changes, raising the possibility that TSP-1's contribution to shear-dependent TGF-β1 activation might be mediated by a thiol-disulfide exchange mechanism. Our data can also provide a novel mechanism for negative feedback control of TGF-β1 activation via depletion of TSP-1 and may reconcile many of the apparently inconsistent observations that have previously been made about the role of TSP-1 in TGF-β1 activation both *in vitro* and *in vivo*.

## Materials and Methods

### Antibodies and Reagents

A murine monoclonal antibody (mAb) to TSP-1 (Ab-11) was obtained from NeoMarkers (Fremont, CA), a chicken polyclonal IgY antibody to human TGF-β1 and recombinant human TSP-1 (rhTSP-1) were obtained from R&D Systems (Minneapolis, MN), TSP-1 inhibitor and control peptides were obtained from Ana Spec Inc. (San Jose, CA), and Streptavidin coupled to magnetic beads was obtained from Invitrogen (Carlsbad, CA). All other reagents and antibodies were from the same sources previously reported[12].

### TGF-β1 Assays

Both immunologic (ELISA) and functional (mink lung epithelial cell; MLEC) assays were used to measure active TGF-β1 before and after treating the samples with 0.2 volume of 1N HCl for 20 min at room temperature to convert latent TGF-β1 to active TGF-β1 as described previously[12].

### Mouse Experiments

#### Mice

A total of 113 wild-type (WT) and 113 TSP-1-null mice (*Thbs1^−/−^*) on a C57Bl/6 background were obtained from Jackson Laboratory (Bar Harbor, ME). Mice were housed in a controlled environment (23±2°C; 12 hr light/dark cycles), and fed a standard diet (5001, Purina Mills; Richmond, IN). All experimental procedures conformed to the recommendations of the Guide for the Care and Use of Laboratory Animals (National Research Council, Washington D.C: National Academy Press; 1996. NIH publication no. 78-23), and approved by The Rockefeller University Animal Care and Use Committee. All mouse experiments were done blindly.

### Hematological data

Hematological parameters were analyzed on EDTA-anticoagulated blood from 16 WT and 16 *Thbs1^−/−^* mice within 30–60 min after blood collection using an automated dual angle light scatter instrument (ADVIA120, Bayer Diagnostics, Tarrytown, NY) as described previously[51]

### Latent TGF-β1 activation in mouse platelet releasates, serum and plasma

In an attempt to simulate *in vivo* platelet deposition, aggregation, and secretion, followed by exposure of the releasate to systemic shear force, WT and *Thbs1^−/−^* mouse releasates were prepared from washed platelets treated with thrombin (0.125 U/mL) without stirring for 5 min at 37°C and then the releasates were subjected to either stirring or shear as described previously[12]. We also analyzed mouse serum prepared as previously described[12], to simulate situations in which *in vivo* thrombin generation results in coagulation. For mouse plasma, blood was drawn percutaneously using an ultrasound-guided left-ventricular puncture and placed in a polypropylene tube containing 0.1 volume of 0.1 M trisodium citrate. Plasma was collected after centrifuging the sample at 3,000×g for 15 min at 4°C immediately after blood collection.

Samples of platelet releasates, serum, and plasma (150−200 µL) were incubated at 37°C with stirring in a glass cuvette (7×50 mm) containing a metal stir bar (1×5 mm) for 2 hours in an aggregometer (Kowa Optimed Inc, Tokyo, Japan) or subjected to a shear rate of 1,800 s^−1^ in a cone and plate device (ImpactR; DiaMed Inc. Miami, FL) at 37°C for the indicated time periods. Active TGF-β1 was measured before and after acidification of samples (to convert latent TGF-β1 to active TGF-β1) using the ELISA and/or MLEC assays. For the stirring experiments, 14 WT and 14 *Thbs1^−/−^* mice were used to prepare platelet releasates, 15 WT and 15 *Thbs1^−/−^* mice were used for rhTSP-1 reconstitution assay, 23 WT and 23 *Thbs1^−/−^* mice were used to prepare serum samples, and 7 WT and 7 *Thbs1^−/−^* mice were used to prepare plasma. For shearing experiment, serum samples from 10 WT and 10 *Thbs1^−/−^* mice were tested.

### Labeling of Free Thiols

Proteins containing free thiols in human platelet releasates were labeled with 3-(N-maleimidylpropionyl) biocytin (MPB) as described previously[12], [52]. Briefly, MPB (100 µM final concentration) was added to platelet releasates and the samples were either stirred at 1200 rpm or subjected to a shear rate of 1800 s^−1^ for the indicated time periods at 37°C. Residual unreacted MPB was quenched by adding reduced glutathione (GSH; 200 µM). In some studies, MPB-labeled proteins were incubated with Streptavidin-coated magnetic beads (Dynabeads, Invitrogen) to isolate the MPB-labeled proteins. The beads were washed and then resuspended in Tris-glycine SDS sample buffer (Invitrogen) to release the proteins from the beads; the solution was heated to 100°C for 3 min and electrophoresed in 8−16% gradient Tris-glycine gels (Invitrogen). After transfer of proteins to polyvinylidene fluoride (PVDF) membranes, biotinylated proteins were detected with streptavidin-horse radish peroxidase (HRP) using chemiluminescence (ECL detection system, Amersham; Pittsburgh, PA). TSP-1 was detected with anti-TSP-1 mAb (Ab-11). Appropriate secondary antibodies conjugated to horse radish peroxidase (HRP) (Jackson Laboratories; West Grove, PA) were used to detect bound antibodies by chemiluminescence. Band intensities were quantitated using image analysis software (NIH-Scion Image; Scion Corporation; Frederick, MD).

### Mass spectrometic analysis

MPB-labeled proteins were incubated with Streptavidin-coated magnetic beads (Dynabeads, Invitrogen) to isolate the MPB-labeled proteins. The beads were washed and then resuspended in Tris-glycine SDS sample buffer (Invitrogen, Carlsbad, CA) to release the proteins from the beads; the solution was heated to 100°C for 3 min and electrophoresed in 8–16% gradient Tris-glycine gels. The gel band at Mr approximately 450 kD was excised, diced, and treated with 10 mM DTT in 0.1 M ammonium bicarbonate to reduce disulfide bonds. Free cysteine residues were alkylated with freshly made 55 mM iodoacetic acid in 0.1 M ammonium bicarbonate. Tryptic digestion [25 ng/µl of sequence grade-modified trypsin (Promega) in ammonium bicarbonate buffer] was conducted for at least 16 h at 30°C. The resultant tryptic peptide mixture was separated by gradient elution with a nano-HPLC system (Dionex, Sunnyvale, CA) interfaced with a LTQ-Orbitrap mass spectrometer (Thermo Fisher Scientific Inc). The MS/MS spectra were analyzed with MASCOT search engine to identify proteins from primary sequence databases.

### Latent TGF-β1 activation in vivo in the carotid artery thrombosis model

Mice (28 WT and 28 *Thbs1^−/−^*) were anesthetized with isoflurane and maintained under inhalation anesthesia as previously described[53]. Mouse body temperature was monitored with a rectal probe and thermistor (THM100; Indus Industries, Houston, TX) and maintained at 37±2°C with a temperature-controlled heating pad (THM100). The left common carotid artery was dissected and a Doppler flow probe (Transonics 0.5; Transonic Systems, Ithaca, NY) was placed on the carotid artery proximal to the bifurcation as previously described[54]. Thrombosis was induced by placing a 1×3 mm piece of filter paper (Whatman no. 1; Maidstone, UK) saturated with either 8 or 20% FeCl_3_ on the exposed segment of the carotid artery for 3 min. Occlusive thrombi formed uniformly within approximately 5 min after exposure to FeCl_3_ and after an additional 5 or 120 min, approximately 4 mm arterial segments containing the thrombi were excised. The segments were then opened, and the thrombi were removed and dispersed in 150 µL of HBMT buffer, pH 7.4 on ice for 1 hour. After centrifugation at 14,000×g for 20 min at 4°C, the carotid extracts were saved and the pellets were lysed with 150 µL of buffer containing 1% Triton X-100 for 1 hour at 4°C and diluted with 150 µL of HBMT buffer, pH 7.4. Lysates were collected after centrifugation at 14,000×g for 20 min at 4°C. Total TGF-β1 in the extracts and lysates were measured by ELISA. Active and total TGF-β1 in the extracts from the carotid artery thrombi were also measured by the more sensitive MLEC biological assay. To make sure that only TGF-β1 activity was being measured in this assay, samples were measured in the presence or absence of an anti-TGF-β1 neutralizing antibody (R&D Systems) and only the neutralizable activity was reported. We found that 50−80% of active TGF-β1 was inhibited by the anti-TGF-β1 neutralizing antibody. To estimate the numbers of platelets contained in the thrombi, carotid lysates were immunobloted for αIIb using a goat anti-αIIb goat polyclonal antibody (SantaCruz Biotechnology, CA) and compared with lysates prepared from known numbers of washed murine platelets. In preliminary experiment, we assessed whether FeCl_3_ (100 µM) could directly activate TGF-β1 in WT platelet releasate samples and found only a minimal increase in active TGF-β1 (active TGF-β1 increased from 0.24% to 0.30% of total TGF-β1 after 2 hours of incubation at 37°C).

### Statistical Analysis

Ranges are given as mean±SD. S-PLUS 8.0 (www.insightful.com, Seattle, WA) was used for graphics and analysis. A mixed model with conditions (stirring/shearing versus unstirred/unsheared, or 5 min versus 120 min, respectively) and mouse strain (WT versus *Thbs1^−/−^*) as fixed factors and date of experiments and animal within date of experiment as nested random factors were used for ANOVA. The condition versus strain interaction was tested to assess differences in responses between strains. Since combining experiments into a single analysis when different interventions have similar effects increases sample size and, thus, power, whereas combining them when the interventions have different effects increases the within-group variance and, thus, avoids the risk of reporting false positive results. Hence, we conservatively combined data obtained under similar condition (stirring and shear, complete occlusion obtained under different concentrations of clotting agents) by adding an additional (fixed) stratification factor to the ANOVA model, where appropriate. Comparisons between WT and *Thbs1^−/−^* mice for individual time points across experimental conditions (date of experiment) by means of Student's t-test for independent samples were also included, with the recognition that this analysis does not reflect baseline conditions and environmental confounders.
